# Molecular Mimicry: a Paradigm of Host-Microbe Coevolution Illustrated by *Legionella*

**DOI:** 10.1128/mBio.01201-20

**Published:** 2020-10-06

**Authors:** Sonia Mondino, Silke Schmidt, Carmen Buchrieser

**Affiliations:** a Institut Pasteur, Biologie des Bactéries Intracellulaires, Paris, France; b CNRS UMR 3525, Paris, France; c Sorbonne Université, Collège Doctoral, Paris, France; University of Texas Health Science Center at Houston

**Keywords:** molecular mimicry, eukaryotic-like proteins, *Legionella*, amoeba-resistant bacteria, host-pathogen interactions, *Legionella pneumophila*

## Abstract

Through coevolution with host cells, microorganisms have acquired mechanisms to avoid the detection by the host surveillance system and to use the cell’s supplies to establish themselves. Indeed, certain pathogens have evolved proteins that imitate specific eukaryotic cell proteins, allowing them to manipulate host pathways, a phenomenon termed molecular mimicry. Bacterial “eukaryotic-like proteins” are a remarkable example of molecular mimicry. They are defined as proteins that strongly resemble eukaryotic proteins or that carry domains that are predominantly present in eukaryotes and that are generally absent from prokaryotes. The widest diversity of eukaryotic-like proteins known to date can be found in members of the bacterial genus *Legionella*, some of which cause a severe pneumonia in humans. The characterization of a number of these proteins shed light on their importance during infection. The subsequent identification of eukaryotic-like genes in the genomes of other amoeba-associated bacteria and bacterial symbionts suggested that eukaryotic-like proteins are a common means of bacterial evasion and communication, shaped by the continuous interactions between bacteria and their protozoan hosts. In this review, we discuss the concept of molecular mimicry using *Legionella* as an example and show that eukaryotic-like proteins effectively manipulate host cell pathways. The study of the function and evolution of such proteins is an exciting field of research that is leading us toward a better understanding of the complex world of bacterium-host interactions. Ultimately, this knowledge will teach us how host pathways are manipulated and how infections may possibly be tackled.

## Abstract (precis)

Through coevolution with host cells, microorganisms have acquired mechanisms to avoid the detection by the host surveillance system and to use the cell’s supplies to establish themselves. Indeed, certain pathogens have evolved proteins that imitate specific eukaryotic cell proteins, allowing them to manipulate host pathways, a phenomenon termed molecular mimicry. Bacterial “eukaryotic-like proteins” are a remarkable example of molecular mimicry. They are defined as proteins that strongly resemble eukaryotic proteins or that carry domains that are predominantly present in eukaryotes and that are generally absent from prokaryotes.

## INTRODUCTION

During host-pathogen interactions, a continuous coevolution of the host defenses and the microbes’ mechanisms of evasion take place. In some pathogens, this process led to the evolution of secreted effector proteins that imitate eukaryotic functions, so-called molecular mimics. The concept of molecular mimicry was initially described by Raymond T. Damian in 1964, and it referred to the sharing of antigenic determinants between parasite and host (1). He discussed the origin and consequences of molecular mimicry, showing that it allows the parasite to avoid recognition by the host immune system and thus to survive. However, due to the resemblance between epitopes from the microorganisms and the antigens present in the host, infections can initiate or stimulate a strong autoimmune response. Hence, it was suggested that molecular mimicry may also be involved in the development of human autoimmune diseases (2; reviewed in reference 3).

Later, the concept of molecular mimicry was expanded, referring to the display of pathogen-encoded factors that resemble structures of the host at the molecular level and that benefit the pathogen due to this resemblance. These mimics can be perfect mimics when they co-opt host factors or imperfect when they resemble host components and yet perform distinct functions which confer an advantage to the pathogen (4, 5). Four different types of mimicry exist: (i) similarity in the sequences and structures of full-length proteins or domains, (ii) structural similarity without sequence homology, (iii) similarity in protein short linear motifs (SLiMs), also known as motif mimicry, and (iv) similarity of binding surface architectures even without sequence homology, known as interface mimicry (6, 7).

Molecular mimics can emerge by two main mechanisms. The first one is convergent evolution, in which nonhomologous proteins encoded by microorganisms evolve features of host proteins mainly through mutations (5, 8). These microbial proteins then mimic specific chemical groups or biophysical properties of host proteins that are relevant for protein function. However, such mimics are difficult to detect, as detailed functional and structural analyses are necessary (5, 8). Alternatively, mimics can arise from the acquisition of host cell genes through horizontal gene transfer, which can be evidenced by sequence similarity with host proteins and by phylogenetic analyses. After horizontal acquisition, genes are generally sculpted and evolve further to be efficiently processed by the bacterial transcriptional/translational machinery. This may involve the loss of introns, the acquisition of regulatory elements necessary for correct gene expression, and the acquisition of a secretion signal that will allow recognition of the acquired protein by bacterial secretion systems. As a consequence, the divergent evolution of the mimic may obscure the common origins of these genes (5). Convergent evolution and vertical acquisition of eukaryotic-like functions are not exclusive processes but can be observed even in a single bacterial protein. An interesting example is SptP, a type III secreted effector of Salmonella enterica serovar Typhimurium with N-terminal GTPase-activating protein (GAP) activity for Rac1 and Cdc42 (9). The crystal structure of the SptP-Rac1 complex revealed that the GAP domain functionally mimics host GAPs through a combination of specific structural elements, suggesting that the GAP activity has evolved convergently from the selective pressures of host-pathogen coevolution (10). In contrast, the carboxyl-terminal part of SptP, with tyrosine-phosphatase activity, harbors sequence and structure similarity to those of their eukaryotic counterparts, suggesting that this function was acquired by horizontal gene transfer (8). Thus, horizontal gene transfer followed by fusion to a bacterial protein might be an important mechanism for the emergence of bacterial proteins with eukaryotic-like domains (11).

The increasing availability of bacterial genome data improved our knowledge of the diversity of bacterial mimics that resemble eukaryotic proteins or that harbor eukaryotic-like domains. The latter can be defined according to their distribution within the bacterial or eukaryotic genomes. In a recent study, our group has defined a eukaryotic domain as one that is found in more than 75% of eukaryotic genomes and less than 25% of prokaryotic genomes (12). While these domains tend to be widespread in eukaryotes, they are generally absent or rarely present in bacteria, except in those that closely interact with a eukaryotic host (13). Furthermore, eukaryotic-like proteins, which are bacterial proteins that are similar to eukaryotic proteins (e.g., they have at least 20% amino acid identity over more than a third of the protein length), have been identified in some bacterial genomes (12). The presence of bacterial eukaryotic-like proteins is a strong indicator that such proteins are effectors, which are generally secreted by dedicated secretion systems during infection (14, 15). Indeed, eukaryotic-like proteins and their role in infection have been described in several intracellular pathogens, such as *Legionella*, *Coxiella*, *Mycobacterium*, *Chlamydia*, and *Bacillus* (16–20). However, to our knowledge, the highest number and widest variety of eukaryotic-like proteins have been found in bacteria belonging to the genus *Legionella* (12, 21). Analyses of the *Legionella* genus genome identified proteins encoding 137 different eukaryotic-like domains and more than 200 eukaryotic-like proteins, constituting a remarkable example of molecular mimicry of host proteins by an opportunistic human pathogen (12). In this review, we take *Legionella* as a model and we discuss examples of molecular mimicry of eukaryotic domains and eukaryotic proteins in the context of pathogenesis and symbiosis, with an emphasis on effectors for which a function has been described.

## *LEGIONELLA*, A BLIND COPYCAT

When our group sequenced and analyzed the Legionella pneumophila strain Paris genome, it was surprising to find a high number of genes coding for eukaryotic-like proteins or proteins with eukaryotic-like motifs (15). We thus hypothesized that these proteins may help the bacterium to hijack the host cell (15). *Legionella* spp. are Gram-negative, facultative, intracellular bacteria that are ubiquitously present in aquatic environments. An exception is Legionella longbeachae, which is isolated mainly from moist soil and potting mixes (22, 23). The bacteria are found either free-living, associated with biofilms, or parasitizing protozoan hosts, such as amoebae (24). Apart from its natural protozoan hosts, *Legionella* is also able to infect human alveolar lung macrophages. This infection can lead to a severe pneumonia called Legionnaires’ disease, which can be fatal if not treated promptly (23, 25).

Infection of humans occurs through inhalation of aerosolized, contaminated water droplets generated by artificial water systems, such as air-conditioning units, shower heads, or cooling towers. The number one causative agent of Legionnaires’ disease worldwide is L. pneumophila, causing 80 to 90% of the confirmed cases, followed by L. longbeachae, which is particularly prevalent in Southeast Asia, Australia, and New Zealand (23). Nearly everything that we know today about the intracellular lifestyle of *Legionella* was obtained by studying L. pneumophila. Starting with cell contact, L. pneumophila secretes into the host cytoplasm effectors that help (i) to avoid phagolysosomal degradation and (ii) to establish a dedicated replicative niche, named the *Legionella-*containing vacuole (LCV). These effectors have been shown to manipulate diverse cellular signaling and vesicular trafficking pathways to recruit endoplasmic reticulum (ER)-derived vesicles to the LCV, to redirect cellular proteins to the LCV for nutrient supply, to subvert the host cell immune response, or to suppress autophagy and apoptosis of the infected cell (26).

**FIG 1:**
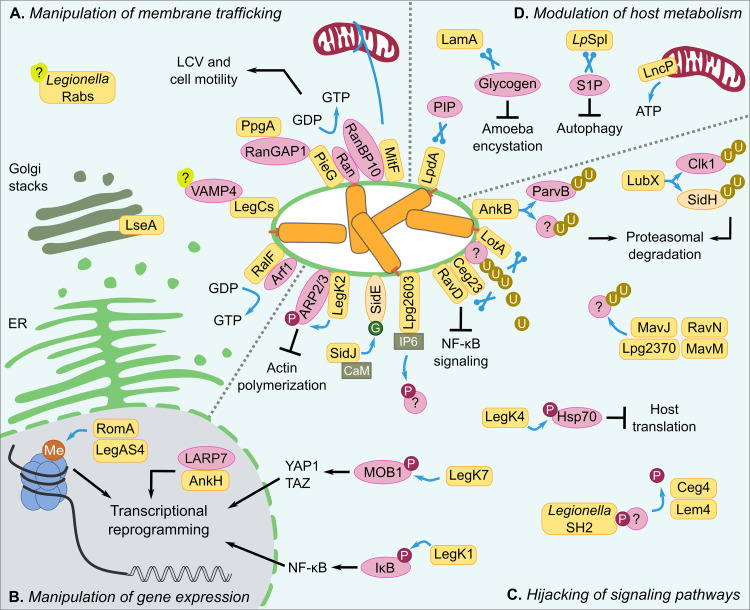
Selected *Legionella* eukaryotic-like T4SS-dependent effectors as discussed herein. *Legionella* spp. translocate eukaryotic-like proteins to manipulate specific host cell processes, like membrane trafficking (A), gene expression (B), signaling pathways (C), and host metabolism (D). While many of these translocated effectors harbor eukaryotic-like domains, others resemble eukaryotic proteins themselves. The functions of the different *Legionella* effectors implicated in the depicted processes are further discussed in the text. Orange box, *Legionella* effector; pink oval, host target protein/molecule; orange oval, *Legionella* target protein; Me, methylation; U, ubiquitination; P, phosphorylation; G, glutamylation, CaM, calmodulin; IP6, inositol hexakisphosphate; ER, endoplasmic reticulum.

An astonishing number (18,000) of different effectors have recently been predicted in the genus *Legionella*, but only 8 of them are conserved in the 58 species analyzed (12). To date, L. pneumophila is the uncontested champion in the bacterial kingdom, with over 330 translocated effectors (representing 10% of its genome) that are delivered to the host cell cytosol through the Dot/Icm type 4B secretion system (T4SS) (27). A large body of research that sheds light on the functional roles of specific effectors encoded by L. pneumophila has been collected in the last several years. These studies have substantially contributed to our understanding of molecular mimicry of eukaryotic protein domains/motifs as a central survival mechanism of *Legionella* (Table 1; Fig. 1).

**TABLE 1:** Eukaryotic-like effectors of *Legionella* with functions discussed herein

Effector(s)	Domain(s)	Function(s)	Identified target(s)	Reference(s)
*Legionella* proteins with eukaryotic domains				
Manipulation of small GTPases and membrane trafficking				
RalF	Sec7	Acts as an Arf1 GEF	Arf1	31, 32
*Legionella* Rab GTPases	Rab GTPase-like	Unknown	Unknown	12, 85
MitF (LegG1)	RCC1	Activates Ran, promotes LCV and host cell motility; implicated in mitochondrial fragmentation	RanBP10	36, 37, 38, 40
PieG	RCC1	Activates Ran, promotes LCV and host cell motility	Ran, RanGAP1	40
PpgA	RCC1	Activates Ran, promotes LCV and host cell motility	RanGAP1	40
LseA	Qc-SNARE	Probably mediates membrane fusion events	Qa-, Qb- and R-type SNAREs	43
YlfB (LegC2), LegC3, YlfA (LegC7)	Coiled-coil motifs reminiscent of Q-SNARE	Modulate membrane fusion events	VAMP4	48, 49, 50, 51

Manipulation of host cell transcription				
RomA	Ankyrin repeat, SET	Changes histone marks; methylates nonhistone proteins	H3K14, AROS	56, 57
LegAS4	Ankyrin repeat, SET	Changes histone marks	H3K4	58
AnkH	Ankyrin repeat	Interferes with transcriptional elongation by RNA polymerase II	LARP7 of the 7SK snRNP complex	61

Manipulation of the ubiquitination pathway				
LubX	U-box	E3 ligase	Clk1, SidH	66, 67, 69
AnkB	Ankyrin repeat, F-box	Interacts with the ubiquitination machinery, supplies amino acids to the *Legionella* vacuole	Skp1, ParvB	72, 73, 75
RavN	U-box-like motif	E3 ligase	Unknown	70
Lpg2370	RING-type E3 ligase	E3 ligase	Unknown	70
MavM	RING-type E3 ligase	E3 ligase	Unknown	70
MavJ	HECT-type E3 ligase	E3 ligase	Unknown	70
LotA	Ovarian tumor (OTU) superfamily of cysteine proteases	Deubiquitinase	Ubiquitin moieties on the LCV	82
Ceg23	Ovarian tumor (OTU) superfamily of cysteine proteases	Deubiquitinase	K63-linked polyubiquitin on the LCV	83
RavD	OTULIN-like interaction surface, papain fold	Deubiquitinase	M1-linked linear ubiquitin on the LCV	84

Manipulation of protein phosphorylation				
LegK1	STPK	Mimics IκB kinases, activates NF-κB signaling	IκB family of inhibitors	88
LegK4	STPK	Inhibits host translation	Hsp70 chaperone family	89, 90
LegK2	STPK	Inhibits actin polymerization on the *Legionella* vacuole	ARPC1B and ARP3 subunits of the ARP2/3 complex	87, 91
LegK7	Structural homology to STPK	Mimics MST1, hijacks the Hippo signaling pathway	MOB1A	92, 93
SidJ	Structural homology to STPK	Pseudokinase that mediates protein polyglutamylation, activated by host calmodulin	SidE family of effectors	94, 95, 96, 97
Lpg2603	Atypical protein kinase structure	Active kinase *in vitro*, activated by host inositol hexakisphosphate	Unknown	98
Ceg4	HAD	Phosphotyrosine phosphatase, putative regulator of MAPK signaling	p38 MAPK	101
Lem4	HAD	Phosphotyrosine phosphatase	Unknown	102
*Legionella* SH2 domain-containing proteins	SH2	Bind to phosphotyrosines	Unknown	104
				
*Legionella* proteins that resemble eukaryotic-like proteins				
*Lp*Spl	Sphingosine 1-phosphate lyase	Restrains autophagy	Intracellular sphingosine	105, 106
LpdA	Phospholipase D	Modulates phosphatidic acid cellular levels	Lipid substrates	107, 108
LncP	Mitochondrial carrier family of proteins	Mediates the unidirectional transport of ATP	ATP	109
Lpg1905, Lpg0971	ecto-NTPDase	Unknown	Unknown	110, 112, 113
GamA	Amylase	Unknown	Unknown	115
LamB	Amylase	Unknown	Unknown	116
LamA	Amylase	Blocks amoeba encystation	Intracellular glycogen	117

### Intracellular lifestyle: *Legionella* effectors that manipulate small GTPases and membrane trafficking.

*Legionella* critically depends on the establishment of the LCV and the hijacking of cellular pathways to efficiently replicate inside the host cell. Small GTPases of the Arf, Rho, Ras, and Rab families play a central role in eukaryotic intracellular trafficking, cell motility, and intracellular signaling events, among others (28). A hallmark of small GTPases is the switch between an active GTP-bound state and an inactive GDP-bound state. The switch between these states is mediated by guanine nucleotide exchange factors (GEFs), which induce the exchange of GDP for GTP, leading to GTPase activation, and GTPase-activating proteins (GAPs), which accelerate the hydrolysis of GTP, leading to the inactive state. Small GTPases can be activated only by GEFs when they are bound to membranes, which requires the constant cycling of small GTPases between endomembranes and the cytosol, which is regulated by solubilizing guanine dissociation inhibitors (GDIs) (28). These eukaryotic/endogenous regulators of small GTPases interact with the proteins via a shared conserved domain. Arf GEFs contain Sec7 domains and regulate protein and lipid trafficking in eukaryotic cells (29). Human regulator of chromatin condensation 1 (RCC1) contains signature RCC1 repeats and functions as a GEF for the small GTPase Ran (30).

L. pneumophila uses the molecular mimicry of all of the above-mentioned eukaryotic protein domains, resulting in the bacterial manipulation of membrane trafficking. The translocated effector RalF contains a Sec7 domain and acts as an Arf GEF in the host cell, directly activating and recruiting Arf1 to the LCV membrane (31). Indeed, structural analysis confirmed that the Sec7 domain of RalF has the same overall structure as its eukaryotic counterparts, demonstrating that the bacterial and eukaryotic proteins are structurally highly conserved (32).

Furthermore, L. pneumophila uses at least six secreted effectors (SidM [DrrA], SidD, AnkX, Lem3, LepB, and LidA), which mediate the recruitment of Rab1 and its functional modification to facilitate the fusion of ER-derived vesicles with the LCV membrane (for reviews on this topic, see references 33 and 34). Surprisingly, most of these Rab1-modulating effectors are absent in *L. longbeachae* and several other *Legionella* species (12). Notably, genome analyses identified genes encoding eukaryotic Rab GTPase domain-containing proteins in some *Legionella* species, including *L. longbeachae*, suggesting that these bacterial Rab-like proteins are able to subvert host cell trafficking (12). Although there is no evidence to date that these effectors functionally replace the Rab1-modulating proteins of L. pneumophila or that they use the canonical GTP/GDP cycle, they are an intriguing example of molecular mimicry in the genus *Legionella*, and further research will shed light on the specific roles of these unique bacterial Rab-like proteins in infection.

Another eukaryotic-like domain used by L. pneumophila is the RCC1 domain, a seven-beta-propeller fold specific for Ran GEFs, which was first identified in the secreted effector MitF (LegG1) (35). In fact, MitF (LegG1) has been shown to function as a bacterial Ran activator, promoting microtubule stabilization, LCV motility, and cell migration during infection (36, 37). In addition, it was recently shown that MitF (LegG1) is implicated in mitochondrial fragmentation, probably through WASP-Arp2/3-mediated recruitment of DNM1L. This modulation of mitochondrial dynamics by L. pneumophila induces a Warburg-like metabolism in the host cell that promotes bacterial replication (38). Several intracellular pathogens induce a shift in the host cell metabolism, probably supporting the bacterium’s specific nutritional needs during infection (39). A recent study highlights an interesting history of divergent evolution of RCC1-containing effectors among different L. pneumophila strains, ultimately leading to changes of their specific cellular targets but not their Ran GEF function. The RCC1 repeat-containing proteins MitF (LegG1) and PpgA of L. pneumophila strain Philadelphia-1 target RanBP10 and RanGAP1, while PieG from L. pneumophila strain Paris activates Ran by binding to Ran itself and RanGAP1 (40). Despite their different cellular targets, all three effectors lead to Ran activation as well as increased LCV and host cell motility (40).

In addition, L. pneumophila secretes effectors that mimic eukaryotic soluble *N*-ethylmaleimide-sensitive factor attachment protein receptors (SNAREs). SNAREs contain coiled-coil domains that consist of amphipathic alpha-helices that can wrap around each other to form helical bundles. These proteins tether membranes together to mediate membrane fusion events in the eukaryotic cell, for example in vesicular trafficking (41, 42). SNAREs are broadly classified into R- and Q-SNAREs based on the interaction of an arginine residue with three glutamine residues in the SNARE core complex. The analysis of LseA in L. pneumophila revealed strong sequence homology to the fungal SNARE protein syntaxin-6 (43). LseA resembles a Qc-SNARE protein by its coiled-coil domain, and it contains a C-terminal eukaryotic CAAX motif predicted to be necessary for binding to host organelles. C-terminal prenylation motifs, or CAAX boxes, harbor a conserved cysteine residue that is lipidated by host prenylation enzymes during infection (44–46). Indeed, it was shown that LseA localizes to Golgi membranes and binds multiple host SNAREs of the Qa, Qb, and R types, thus probably mediating membrane fusion events in Golgi membrane-associated pathways (43). The C-terminal CAAX prenylation motif, like nuclear localization signals (NLS) or ER retention motifs, are defined as SLiM mimicry, which allows to position effectors to specific host organelles. SLiM mimicry has recently been reviewed extensively (47).

The putative Q-SNAREs LegC3 and YlfA (LegC7) were identified by sequence-based structure prediction to contain coiled-coil domains and were shown to disrupt yeast endosomal trafficking (48, 49). Moreover, these two proteins together with YlfB (LegC2), another SNARE-like protein, were shown to mediate membrane fusion events by binding to the eukaryotic R-SNARE vesicle-associated membrane protein 4 (VAMP4), possibly promoting LCV expansion (50, 51).

Taken together, molecular mimicry of Sec7, Rab GTPase, coiled-coil, or RCC1 eukaryotic domains facilitates L. pneumophila intracellular replication through the modulation of proteins that are key components of eukaryotic membrane trafficking pathways.

### Targeting the control center: *Legionella* effectors that manipulate host cell transcription.

During infection, some of the secreted bacterial proteins, so-called nucleomodulins, ultimately hijack the cell nucleus (52, 53). Examples of eukaryotic mimics that modulate the transcriptional machinery of the host cell include bacterial proteins containing ankyrin motifs and SET [eukaryotic su(var)3-9, enhancer-of-zeste and trithorax] domains. Ankyrin repeats mediate protein-protein binding, while SET domains mediate methylation of lysine residues of histones and modify chromatin condensation as well as transcriptional activity at specific sites (54, 55). The T4SS-dependent effector RomA of the L. pneumophila strain Paris contains two eukaryotic-like domains: a C-terminal ankyrin repeat domain and an N-terminal SET domain. RomA methylates lysine 14 of histone H3 (H3K14), a residue usually acetylated at active promoters, to downregulate gene transcription in infected cells, including genes associated with innate immunity (56). In addition, it was recently shown that RomA also methylates nonhistone proteins in human cells, suggesting a multifaceted role for this effector during infection. RomA also targets AROS, a regulator of the human deacetylase SIRT1, although the specific role of this modification during *Legionella* infection remains to be investigated (57). LegAS4, the homolog of RomA in L. pneumophila strain Philadelphia-1, was reported to localize to the cell nucleolus, where it methylates H3K4, probably leading to increased transcription of host cell ribosomal DNA (58). However, as with infection of alveolar epithelial cells with L. pneumophila strain Paris, infection with L. pneumophila strain Philadelphia-1 leads to strong methylation of the H3K14 histone mark (59). Further structural analysis revealed that the LegAS4 SET domain is structurally similar to the eukaryotic SET domain, suggesting that this protein may have evolved from a common eukaryotic ancestor by horizontal gene transfer (60).

AnkH, an ankyrin repeat-containing protein, is the only effector known thus far for which a direct role of the ankyrin repeat has been described in *Legionella* infection. It is also one of eight core effectors common to all *Legionella* species sequenced to date (12, 21). L. pneumophila AnkH directly interacts with the nuclear protein La-related protein (LARP7), which is a component of the 7SK small nuclear ribonucleoprotein (snRNP) complex in human cells. The AnkH-LARP7 interaction partially impedes interaction of LARP7 with the 7SK snRNP complex components, thus interfering with transcriptional elongation by RNA polymerase II. As a consequence, AnkH-dependent global transcriptional reprogramming of the host cell promotes intracellular bacterial growth (61). The N-terminal domain of AnkH contains four ankyrin repeats with a typical structural fold of two alpha-helices, forming a helix-turn-helix motif, joined by a beta-hairpin loop. It was observed that a mutation of the third beta-hairpin loop in the ankyrin repeat domain abrogates binding of AnkH to LARP7 and leads to an intracellular growth defect of L. pneumophila. Taken together, these findings suggest that AnkH-mediated transcriptional reprogramming is essential for infection (61).

There is an increasing number of bacteria for which molecular mimicry of eukaryotic proteins that target the host cell nucleus has been described. Like *Legionella*, Chlamydia trachomatis, Bacillus anthracis, and Burkholderia thailandensis secrete SET domain-containing proteins that target host cell histones. Furthermore, Anaplasma phagocytophilum secretes an ankyrin repeat-containing protein (AnkA) that directly binds to host DNA, leading to decreased transcription, as reviewed in reference 52. Genome analyses might identify new eukaryotic mimics targeting the host nucleus among *Legionella* effectors, as well as in other bacterial pathogens.

### Forging ubiquitin signaling: *Legionella* effectors that target the cellular ubiquitination pathway.

Ubiquitination is a key posttranslational modification with functions in the degradation of proteins, vesicular trafficking, innate immune response, autophagy, and apoptosis. Protein ubiquitination involves the covalent attachment of ubiquitin to the epsilon amino group of lysine as singular chains or branched chains. Three enzymes mediate this reaction in an ATP-dependent manner: a ubiquitin-activating enzyme (E1), a ubiquitin-conjugating enzyme (E2), and a ubiquitin ligase (E3) that confers target protein specificity (62). Eukaryotic E3 enzymes are broadly classified into two classes: HECT and RING-type E3 ligases. The RING E3 enzymes are characterized by their RING or U-box fold catalytic domain, and some multicomponent RING E3 complexes also include F-box domain-containing proteins (63). RING and U-box domains share overall structural similarity; however, U-box domains do not contain central Zinc-binding residues found in RING domain proteins (64). RING/U-box E3 ligases function as scaffolds for transfer of ubiquitin to target proteins without the attachment of ubiquitin to the E3 ligase itself, whereas HECT-like E3 ligases form a covalent thioester E3-ubiquitin intermediate via a conserved cysteine in their active site before transfer of ubiquitin to the target protein (64, 65).

Many bacterial pathogens, including *Legionella*, *Salmonella*, and *Shigella*, target the cellular ubiquitination machinery (65). Interestingly, particular amoeba-associated bacteria encode a large number of proteins that most likely interfere with the host’s ubiquitination signaling, suggesting that it is crucial for bacterial replication in protozoan hosts (13). Indeed, nearly all *Legionella* genomes analyzed to date contain F-box (1 to 18 per genome)- and/or U-box (1 to 3 per genome)-encoding genes (12). The T4SS effector LubX contains two U-box domains, and it was shown to mediate polyubiquitination of the host Cdc2-like kinase 1 (Clk1); however, the consequence of this modification needs to be further elucidated (15, 66). In addition, LubX was shown to target the *Legionella* effector SidH for proteasomal degradation at late stages of infection, thus functioning as a key regulator to temporally coordinate the activity of a cognate effector (67). Thus, LubX was the first identified metaeffector, meaning effectors that target another effector during infection. Subsequently, it was found that putative metaeffectors are present in considerable numbers in the *Legionella* genomes (68). Interestingly, functional and structural analyses revealed that only the N-terminal U-box domain of LubX has E3 ligase activity and that it is the exclusive E2-interacting module within the protein (69). The second C-terminal U-box, however, seems to be necessary for the interaction of LubX with the target proteins, although the single residue that appears to be critical for binding of LubX to the cognate effector SidH was found to be located outside the U-box fold (66, 69).

Despite a lack of sequence homology to known eukaryotic E3 ligases, the crystal structure of the N-terminal region of RavN revealed a U-box-like motif with a surface area analogous to those of the E2 binding regions of other eukaryotic E3 ligases (70). This suggests that RavN has been acquired by *Legionella* through horizontal gene transfer early during evolution and structurally altered over time in order to best fulfil its current function. Indeed, RavN was shown to function as an E3 ubiquitin ligase in eukaryotic cells (70). Using pairwise comparison of profile hidden Markov models, three other *Legionella* E3 ligase effectors with homology to RING-type E3 (Lpg2370 and MavM) or to HECT-type E3 ligases (MavJ) were identified, all of which exerted ubiquitinase activity in transfected cells (70).

The L. pneumophila genomes sequenced to date encode one to four F-box domains each (12, 15). The functionally best-described F-box protein is AnkB, which also contains a eukaryotic ankyrin domain and, in certain strains, also a CAAX motif (71). AnkB interacts with Skp1 and the host ubiquitination machinery to modulate the ubiquitination status of ParvB, a linker of cytoskeletal dynamics and cell survival (72, 73). In addition, AnkB of L. pneumophila strain Philadelphia-1 was shown to be prenylated by the host prenyltransferase machinery at the CAAX motif to anchor the protein in the LCV membrane (44). It was recently proposed that AnkB of L. pneumophila strain Paris, which does not contain a CAAX box, harbors an ER retention motif instead. This motif may allow this protein to be anchored in the LCV membrane (74). It has been suggested that AnkB mediates degradation of polyubiquitinated proteins on the LCV that are further used by L. pneumophila as nutrients (75).

In addition to having proteins with the eukaryotic U-box and F-box domains, *Legionella* translocates proteins that manipulate ubiquitination signaling through noncanonical mechanisms of ubiquitination. Members of the SidE family of effectors attach phosphoribosylated ubiquitin to a serine residue of host Rab GTPases in an NAD^+^-dependent manner. This previously unknown protein ubiquitination is mediated solely by the *Legionella* effector, independently of host E1 and E2 enzymes (76, 77). It was also shown that SidE family effectors are able to cleave the most common ubiquitin chains found in eukaryotic cells through an N-terminal deubiquitinase domain *in vitro* (78). Based on these novel functions, other deubiquitinases specific for phosphoribosylated ubiquitin were recently identified in L. pneumophila (79). However, as several of these proteins do not encode known eukaryotic domains, we will not discuss them in more detail here (for reviews, see references 80 and 81).

In contrast, *Legionella* encodes other proteins with distant homology to the eukaryotic ovarian tumor (OTU) superfamily of cysteine proteases that function as deubiquitinases. An example is LotA, a T4SS-dependent effector that uniquely harbors two catalytic pockets and localizes to the LCV, where it removes ubiquitin moieties. However, its specific function during infection needs to be further elucidated (82). Furthermore, the effector Ceg23 adopts an OTU-like fold despite limited sequence similarity, as revealed by structural analysis of the N-terminal region (83). Ceg23 specifically cleaves Lys63-linked ubiquitin through a Cys-His-Asp catalytic triad and prevents the accumulation of Lys63-polyubiquitin on the LCV. Interestingly, RavD adopts an interaction surface similar to that of the eukaryotic OTU deubiquitinase with linear specificity (OTULIN) when in complex with linear di-ubiquitin. RavD is a cysteine-dependent deubiquitinase with a unique papain-like fold, which uses an unusual Cys-His-Ser catalytic triad to specifically cleave Met1-linked linear ubiquitin chains. In infection, RavD prevents the accumulation of linear ubiquitin chains on the LCV, leading to a downregulation of the host proinflammatory nuclear factor kappa–light-chain enhancer of activated B cell (NF-κB) signaling (84). Taken together, *Legionella* encodes a remarkable diversity of effectors that help to modulate ubiquitin signaling in host cells.

### Rewriting and exploiting of protein phosphorylation: *Legionella* effectors mimicking eukaryotic kinases and phosphatases.

Protein kinases and phosphatases are key players in signal transduction in mammalian cells. Hence, their function is often hijacked by bacterial effectors during infection. While some effectors described for *Legionella* mimic serine/threonine protein kinases (STPK), others contain haloacid dehalogenase (HAD)-like domains or Src homology 2 (SH2) domains (15, 85, 86). Five different proteins in L. pneumophila that show primary amino acid sequence homology to eukaryotic protein kinases have been identified to date, four of which are secreted effectors that have been characterized in more detail (87).

The L. pneumophila eukaryotic-like STPK LegK1 impacts the host NF-κB pathway through functionally mimicking host inhibitor of nuclear factor kappa B (IκB) kinases (88). The activity of LegK1 might be independent of signaling components upstream of host IκB kinases, as it does not require the canonical activation of its kinase domain, indicating that LegK1 might be constitutively active or regulated by means other than phosphorylation of its activation loop (88). Like LegK1, LegK4 is a constitutively active eukaryotic-like STPK that does not depend on phosphorylation of its activation loop but probably on the stabilizing effect of its kinase domain dimerization (89). LegK4 phosphorylates a conserved threonine in the substrate-binding domain of the host Hsp70 chaperone family (90). Due to LegK4-mediated phosphorylation, Hsp70 has reduced ATPase activity and is impeded in its protein refolding capacity, which ultimately leads to a global translation inhibition of the host cell. Altogether, this suggests that LegK4 impacts host translation and the unfolded protein response, which might be beneficial for bacterial replication (90).

The protein kinase LegK2 was initially reported to be involved in the recruitment of ER vesicles to the LCV and in bacterial replication during infection (87). However, it was shown later that LegK2 phosphorylates the ARPC1B and ARP3 subunits of the actin nucleator ARP2/3 complex to inhibit actin polymerization on the LCV. Thereby, it hinders late endosome/lysosome association with the *Legionella* phagosome, contributing to the bacterial escape from the endocytic pathway (91).

LegK7 was recently identified as a bacterial protein kinase with folding homology to eukaryotic protein kinases such as PKAcα, based on profile hidden Markov model structure prediction (92). LegK7 was shown to hijack the conserved Hippo signaling pathway by functionally mimicking the host Hippo kinase MST1. Indeed, LegK7 phosphorylates MOB1A, which in turn leads to the degradation of the cotranscriptional regulators YAP1 and TAZ, thus triggering a signaling cascade that alters the transcriptional landscape of the host cell to promote L. pneumophila replication (92). A recent crystallographic study of the LegK7-MOB1A complex revealed that the N-terminal half of LegK7 is structurally similar to eukaryotic protein kinases and that MOB1A directly binds to the LegK7 kinase domain. LegK7 uses MOB1A as an allosteric activator and as a scaffold for the recruitment and phosphorylation of the downstream substrates (93).

Additionally, L. pneumophila encodes atypical protein kinases that are activated by host cofactors. One such example is SidJ (Lpg2155), a pseudokinase that contains a eukaryotic Ser/Thr protein kinase-like fold but which mediates polyglutamylation of translocated effectors of the SidE family (94, 95). Interestingly, SidJ function is dependent on the host cofactor calmodulin (94–97). Structural analysis of the translocated effector Lpg2603 revealed atypical kinase features. Lpg2603 is an active kinase *in vitro* that is allosterically activated by the host cofactor inositol hexakisphosphate (98). Another effector that is activated by host cofactors is LtpM, a glycosyltransferase that is activated by host phosphoinositides (99). Importantly, *Legionella* spp. also encode a large variety of kinases and phosphatases that target host phosphoinositide (PI) lipids. The roles of these PI kinases and PI phosphatases, including PI binding effectors and PI lipases, have recently been reviewed in detail and will thus not be further discussed in this review (100).

Recently, two *Legionella* effectors harboring HAD-like domains have been identified as eukaryotic-like protein phosphatases. Ceg4 is an atypical HAD-like phosphotyrosine phosphatase implicated in the regulation of host mitogen-activated protein kinase (MAPK)-signaling pathways (101). Similarly, Lem4 was described as a protein tyrosine phosphatase with structural similarity to the murine phosphatase MDP-1 (102). However, the roles of Ceg4 and Lem4 during *Legionella* infection are open questions for future research.

Besides protein kinases and phosphatases, SH2 domains are important phosphotyrosine-binding modules that function as scaffolds for intracellular kinase signaling cascades. SH2 domains are structurally conserved and consist of an antiparallel beta-sheet surrounded by two alpha-helices (103). *L. longbeachae* was the first prokaryote reported to encode eukaryotic SH2 domains (85). Recently, using a structure-guided sequence alignment, 93 putative SH2 domains were identified in 84 *Legionella* proteins (104). SH2 domains in *Legionella* are distinct in amino acid sequence but share both the SH2 domain fold and a conserved phosphotyrosine-binding pocket with their eukaryotic counterparts (104). Interestingly, some *Legionella* SH2 domains bind to phosphotyrosine peptides with greater affinities than known mammalian SH2 domains and thus were termed phosphotyrosine superbinders by the authors of this study. Moreover, the presence of additional eukaryotic-like domains in some *Legionella* SH2-containing proteins suggests that they may be enzymes that modulate specific pathways during infection (104). Further studies are needed to unveil the functions of this diverse set of *Legionella* proteins in the modulation of host cell phosphotyrosine signaling during infection.

## AS CLOSE AS IT GETS: *LEGIONELLA* EFFECTORS THAT RESEMBLE EUKARYOTIC PROTEINS

In addition to revealing modular effectors with eukaryotic domains, genomic analyses of the *Legionella* genus genome revealed the presence of putative bacterial effectors that resemble eukaryotic proteins over more than a third of the protein length (12). The characterization of many of these mimics showed that these proteins encode the same enzymatic activities as their eukaryotic counterparts. Some of these enzymes target specific metabolic pathways in the host cell, conferring an advantage to the bacteria that allows them to use the resources of their host cell to thrive during infection.

### *Legionella* eukaryotic-like proteins that target the host cell metabolism.

Several of the *Legionella* eukaryotic-like proteins identified to date are predicted to function in metabolic pathways of the host cell, probably to scavenge nutrients from the cell to ensure intracellular replication of the bacteria. One example is the L. pneumophila sphingosine 1-phosphate lyase (*Lp*Spl/LegS2). This protein structurally and functionally mimics eukaryotic sphingosine 1-phosphate lyases (105, 106). Recently, it was shown that *Lp*Spl reduces intracellular sphingosine levels of host cells during infection. The manipulation of the host sphingolipid metabolism restrains the autophagic response and promotes the intracellular survival of L. pneumophila (105). Another effector, LpdA, shows homology with eukaryotic phospholipase D enzymes (107). LpdA hydrolyses several lipid substrates, leading to a modulation of the cellular levels of phosphatidic acid and contributing to the virulence of L. pneumophila in mice (108). A protein shown to interfere with ATP transport is LncP. It shows high sequence homology to eukaryotic proteins of the mitochondrial carrier family (MCF) (109). LncP localizes to the mitochondrial inner membrane, from where it mediates the unidirectional transport of ATP. However, the contribution of LncP activity to L. pneumophila intracellular replication and survival remains to be determined (109).

Furthermore, the L. pneumophila genome encodes two ectonucleoside triphosphate diphosphohydrolases (ecto-NTPDases) (15). These enzymes harbor five conserved apyrase regions and catalyze the hydrolysis of nucleoside triphosphates and diphosphates to the monophosphate form (110, 111). L. pneumophila Lpg1905 and Lpg0971 are eukaryotic-like ecto-NTPDases required for optimal intracellular replication and virulence in a mouse model of infection, although the specific functions of these proteins have yet to be elucidated (110, 112, 113). Unlike many other *Legionella* eukaryotic-like proteins, neither of the ecto-NTPDases are translocated into the host cell by the T4SS (113). However, the presence of a putative N-terminal secretion signal and the detection of protein secretion from bacterial cells suggest that they might be localized in the LCV lumen during infection (112, 114).

Amylases are enzymes that catalyze the hydrolysis of starch and glycogen into glucose. The identification of putative amylases in L. pneumophila, together with the fact that these bacteria do not synthesize starch or glycogen themselves, suggests that these proteins might be relevant for infection. L. pneumophila GamA is a eukaryotic-like glucoamylase secreted by the T2SS, although the importance of this protein during infection needs to be further elucidated (115). In contrast, LamB is a *Legionella* amylase required for intracellular replication and virulence in a mouse model of infection (116). However, it is not yet clear if this protein is a secreted effector, as LamB does not contain a T2SS secretion signal and experimental validation of secretion by the T4SS was unsuccessful (116). Finally, LamA was recently described as a T4SS-dependent amylase that catalyzes rapid glycogenolysis in amoebae, blocking amoeba encystation and promoting L. pneumophila proliferation (117).

These effectors highlight the breadth of molecular mimicry exerted by several *Legionella* effectors which skew host cell pathways and facilitate bacterial infection (Table 1; Fig. 1). Given the diversity and the large number of effectors in the genus *Legionella*, exciting discoveries are ahead of us that may identify further mechanisms of host cell modulation *via* bacterial mimicry of eukaryotic cell processes.

## EUKARYOTIC-LIKE PROTEINS SHAPED BY BACTERIUM-PROTOZOAN INTERACTIONS

In the environment, *Legionella* are able to survive and replicate in a wide variety of protozoa, showcasing the most common mechanism of bacterial proliferation (118). The long-lasting coevolution of *Legionella* with its protozoan hosts has distinctly shaped the bacterial genome (12, 21). Many intracellular bacteria undergo genome reduction as a consequence of specialization to the intracellular lifestyle. However, this phenomenon is not observed in amoeba-resistant bacteria like *Legionella* (119). In contrast, it seems that *Legionella* spp. undergo continuous genome expansion as a consequence of gene acquisition by horizontal gene transfer from their protozoan hosts, a phenomenon that was also corroborated by the finding that the ancestral genomes were probably smaller than they are today (12).

Comparative genomics and evolutionary analyses of nearly the entire *Legionella* genus genome revealed that all *Legionella* species have highly dynamic genomes with a diverse effector repertoire of 18,000 predicted proteins, encompassing at least 137 different eukaryotic domains and over 200 different eukaryotic proteins (12). It has already been shown that many of these predicted effectors have a modular structure and encode a combination of different eukaryotic domains (21). Furthermore, a recent study provided insightful evidence that the combined selective pressures of different amoebal hosts drive the evolution of *Legionella* species. These selective pressures ultimately shape the individual diversity of effector repertoires, which are probably related to the type of amoebae that *Legionella* organisms encounter and the frequency of the encounter (120). The origins of several of the eukaryotic-like proteins in the *Legionella* genome were corroborated by several phylogenetic analyses, which demonstrated that these proteins were acquired through horizontal gene transfer from a protist host (11, 12, 15, 85, 86, 121, 122). Thus, *Legionella* constitute one of the best-described examples of eukaryote-to-prokaryote gene transfer. A classic case constitutes the gene encoding the sphingosine 1-phosphate lyase (*spl*) of L. pneumophila (105). Different evolutionary analyses showed that the *spl* gene was acquired by horizontal gene transfer from a protist host, as the closest homologs are those from *Entamoeba* spp., Tetrahymena thermophila, and Paramecium tetraurelia (106, 122). Further homologs of this gene were found in 16 of the 58 *Legionella* species/subspecies analyzed. The phylogenetic analysis of these different *spl* genes showed that they have been acquired and lost several times during the evolution of the genus (122). Interestingly, *Legionella* spp. seem to acquire genes not only from their protozoan hosts but also from plants or fungi, as evidenced by the identification of eukaryotic-like proteins with pentatricopeptide repeats, alliinase, or caleosin domains in some *Legionella* species (12, 85). Thus, genomic exchange between *Legionella* and higher-order organisms also seems to occur (12).

These findings led to the suggestion that amoebae constitute melting pots of evolution, where gene fluxes in multiple directions may happen among amoebae, intracellular bacteria, fungi, and giant viruses, ultimately contributing to the evolution of these different organisms (119, 123). As an example, the ankyrin-containing protein Lpg2416 from L. pneumophila has its only homolog in the Acanthamoeba polyphaga mimivirus, a giant virus infecting *Acanthamoeba*, suggesting that *Legionella* may have acquired this eukaryotic-like protein from giant viruses (11). Additionally, genes can also be exchanged among different amoeba-related bacteria, as it was shown for bacteria of the orders *Legionellales*, *Chlamydiales*, and *Rickettsiales*, adding complexity to the possible evolutionary scenarios (124–126).

**FIG 2:**
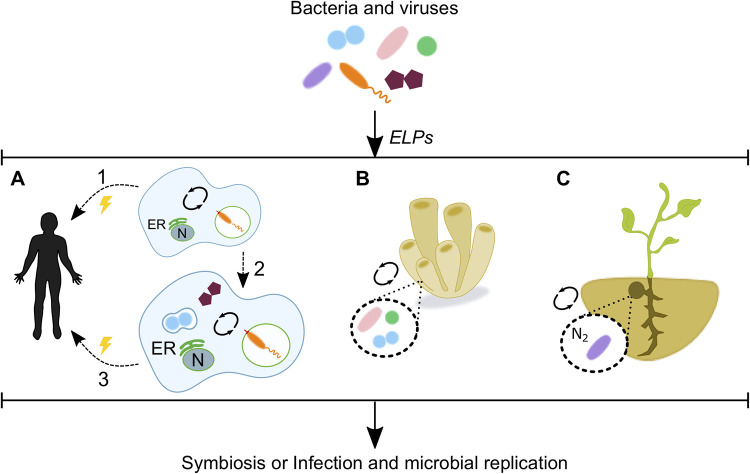
Eukaryotic-like proteins as mediators of pathogenesis and symbiosis. Microorganisms use eukaryotic-like proteins (ELPs) to communicate with their hosts. (A) *Legionella* spp. translocate eukaryotic-like proteins to multiply within protozoa, and this capacity enabled the bacterial transition to humans (arrow 1). The simultaneous occurrence of *Legionella* with other bacteria and giant viruses inside amoebae allows for the genetic interchange between these microorganisms and the host (arrow 2) and the acquisition of diverse functions which might confer an advantage during human infection (arrow 3). (B, C) Despite their role in pathogenicity, eukaryotic-like proteins are also used by cooperative bacterial communities to interact with their hosts. Eukaryotic-like proteins are used by prokaryotes to establish a symbiosis with marine sponges (B) and by rhizobia to form nitrogen-fixing symbioses with legumes (C). N, nucleus; ER, endoplasmic reticulum.

During the last two decades, genome analyses of different bacterium-associated amoebae showed that despite their different lifestyles and phylogenies, they share a set of eukaryotic protein domains necessary for bacterium-host interactions. Indeed, it has been shown that functional domains predominantly found in eukaryotes, such as ankyrin repeats, SEL1 repeats, leucine-rich repeats, and F-box and U-box domains were significantly enriched in the proteomes of amoeba-associated bacteria, like “*Candidatus* Amoebophilus asiaticus” (an obligate intracellular amoeba parasite), *Chlamydiae*, Rickettsia bellii, Francisella tularensis, and Mycobacterium avium (127). Furthermore, phylogenetic analyses were undertaken for some of the identified eukaryotic-like proteins in “*Candidatus* Amoebophilus asiaticus”, suggesting that they had been acquired by horizontal gene transfer from a protozoan host (127). Taken together, these observations suggest that amoebae provide a specific environment for gene exchange between microorganisms invading them as pathogens or symbionts, but also that amoebae might be “active players” in these events as donors of their own DNA (Fig. 2).

It is still unknown how the eukaryotic genes are taken up and how the DNA is subsequently integrated in the bacterial genome. *Legionella* spp., for example, are able to develop competence for natural transformation, which may facilitate the genetic exchange inside amoebae (128). However, other mechanisms, like the transfer of mRNA, may be imagined, in particular as certain *Legionella* genomes contain a gene predicted to encode a group II intron reverse transcriptase (122). Despite the current lack of experimental evidence to corroborate this hypothesis, the nature of this exchange may explain the lack of introns and regulatory elements in the bacterial genes. Once integrated into the bacterial genome, these genes need to evolve to be specifically recognized by the secretion machinery and become secreted proteins. Thus, it has been proposed that a low-level, leaky delivery of these so-called proto-effectors to the host may allow for the selection of mutations to fine-tune the protein’s translocation levels, in order to finally select for an efficient C-terminal translocation signal (27). Recently, it was shown that LvgA of L. pneumophila is an adaptor protein that recognizes effectors for secretion by the T4SS. However, not all effectors were recognized by LvgA, suggesting that the Dot/Icm-type 4B coupling protein (T4CP) complex is likely to be heterogeneous in terms of the adaptors displayed and that additional, yet to be identified adaptor proteins might exist (129).

Taken together, amoebae are key players in the evolution of amoeba-resistant bacteria like *Legionella*. Large-scale genome sequencing of different aquatic amoebae, a field only poorly studied to date, is greatly needed to improve our knowledge of the extent of interkingdom gene transfer and may provide evidence of currently unknown gene fluxes between different species of amoebae and bacteria. This knowledge will further advance our understanding of the origins of bacterial eukaryotic-like proteins and may allow us to elucidate the mechanisms by which they were acquired.

## EUKARYOTIC-LIKE PROTEINS IN THE CONTEXT OF PATHOGENESIS AND SYMBIOSIS

The observation that *Legionella* species are able to multiply within protozoan hosts, primarily aquatic amoebae, led to the idea that the capacity of the bacteria to replicate within human phagocytic cells may have evolved from their ability to survive within protozoa (130), a hypothesis which was later confirmed by different studies (as reviewed in reference 131). Amoebae and macrophages have similar mechanisms of phagocytosis and bacterial inactivation; both consist of degradation of the phagocytized material, further supporting the idea that resistance to amoeba is an important driving force in the evolution of human pathogens. Indeed, pathogenicity might have been an ancient microbial mechanism of defense against predators, which evolved through time to allow microbes to survive within higher organisms (132). Then, amoeba-bacterium interactions drove the selection of bacterial features necessary for life within a eukaryotic host, including the evolution of specific eukaryotic-like proteins (133–135). *Legionella* certainly constitute an example of such adaptations, as it has recently been shown that L. pneumophila growth in macrophages results from the cumulative selective pressures of multiple amoeba hosts, which in some cases lead to redundancy among effectors (120). This observation suggests that different *Legionella* species have evolved distinct virulence mechanisms, including the use of specific eukaryotic-like proteins, as a consequence of their differential adaptations to amoeba predation, which finally enabled the transition from environmental reservoirs to humans (120). However, it is important to point out that eukaryotic-like effectors or effectors with eukaryotic domains might lead to different outcomes in infections of amoeba and human alveolar macrophages. While some effectors may facilitate survival in the environmental host, they may not be beneficial for infection of human cells because they may trigger immune surveillance pathways. One such example is LamA, a translocated amylase, which degrades glycogen in the host cytosol and prevents amoeba encystation. In contrast, the activity of LamA in human macrophages induces an M1-like proinflammatory phenotype of the cells, leading to a growth restriction of the bacteria (117).

Conversely, microbes typically considered beneficial and nonpathogenic can also display features that are typically considered hallmarks of pathogens, suggesting that these gene products may also be mutualistic factors, depending on the context (136). To date, eukaryotic-like proteins have been identified in the genomes of symbiotic bacteria associated with amoebae, fungi, sponges, and plants, suggesting that disease is not the only outcome of microbial molecular mimicry. In contrast, manipulation through molecular mimicry is also beneficial for the establishment of cooperative interactions between bacterial communities and their eukaryotic hosts (13) (Fig. 2). For example, root nodule bacteria (also known as rhizobia) are free-living soil bacteria that have the ability to form nitrogen-fixing symbioses with legumes (137). A multistep analysis of 163 rhizobial genomes identified five domains of eukaryotic origin that were overrepresented in these bacteria, compared to their presence in a negative-control genome set from phylogenetically related organisms not known to be associated with plants. Within these five putative eukaryotic-like proteins, only three were predicted to be secreted, overall suggesting that these rhizobial eukaryotic-like proteins may contribute to the modulation of the plant host responses during the symbiosis establishment (137).

Over the last decade, eukaryotic-like proteins have also been identified in the symbionts of marine sponges. Metatranscriptomic analyses of the microbiomes of three different sponges, namely, *Cymbastella concentrica*, *Scopalina* sp., and Tedania anhelens, identified eukaryotic-like proteins in 2.3%, 1.4%, and 1.3% of all prokaryotic transcripts, respectively, demonstrating constituent expression of eukaryotic-like proteins in the sponge symbionts (138). Particular classes of eukaryotic-like proteins and domains, such as cadherin, tetratricopeptide repeats, and ankyrin repeats, were expressed differently between the microbiomes of the different sponge species. Moreover, some of them were associated or cotranscribed with translocation systems, suggesting their involvement in bacterium-host interactions (138). Indeed, it has been observed that the heterologous expression of four eukaryotic-like, ankyrin-repeat proteins from a sponge symbiont allowed Escherichia coli to modulate phagocytosis by amoebae (139). Hence, the functions of these eukaryotic-like proteins might facilitate bacterial survival and subsequent establishment within the sponge cells. A recent metagenomic analysis showed a high number of genes encoding eukaryotic-like proteins in the microbiomes of two of the most abundant Antarctic sponges, *Myxilla* sp. and Leucetta antarctica, thus pointing to common molecular mechanisms mediating symbiosis with sponges across different environments, including Antarctica (140).

In summary, there is increasing evidence suggesting that modulation of host pathways by eukaryotic-like proteins may constitute a common mechanism associated with bacterial survival within protozoan, fungal, plant, and metazoan hosts. The final outcomes of these interactions are not uniform; instead, bacterial clearance, symbiosis, or disease can occur (Fig. 2).

## FINAL REMARKS

Mimicry constitutes one of the most common adaptive mechanisms in nature, and bacteria do not fall short, since this tool at the molecular level (known as molecular mimicry) represents a key element of bacterium-host interactions. *Legionella* adaptation to protozoan hosts makes these bacteria a remarkable model for the study of molecular mimicry. Indeed, the increasing characterization of *Legionella* proteins that mimic specific eukaryotic functions gives an idea of the multiplicity of host pathways that are targeted during infection. Moreover, the recent identification of eukaryotic-like proteins encoded in the genomes of other amoeba-resistant and -symbiotic bacteria highlights the importance of these eukaryotic-like mimics both in the context of infection and as a means of bacterial communication. In this regard, the identification of bacterial eukaryotic-like proteins in microbiomes is particularly exciting. Overall, eukaryotic-like proteins are powerful tools that allow bacteria to communicate with their hosts and to thrive in the environment. The study of molecular mimics highlights the complexity of bacterium-host interactions and bacterium-host coevolution, particularly important in the context of human health and disease.
